# P11 A quality improvement baseline evaluation of IV gentamicin management in the acute hospital setting following introduction of an electronic prescribing system (HEPMA)

**DOI:** 10.1093/jacamr/dlae217.015

**Published:** 2025-01-23

**Authors:** Emily Simpson, Rachael Rodger, Hiera Qadri

**Affiliations:** Pharmacy Department, Royal Alexandra Hospital, NHS Greater Glasgow & Clyde, Scotland; Pharmacy Department, Royal Alexandra Hospital, NHS Greater Glasgow & Clyde, Scotland; Pharmacy Department, Royal Alexandra Hospital, NHS Greater Glasgow & Clyde, Scotland

## Abstract

**Background:**

Gentamicin is commonly prescribed IV in the empirical management of infections. It has a narrow therapeutic index requiring therapeutic drug monitoring (TDM) to optimize efficacy and minimize patient risk of renal and ototoxicity.^1^ In NHS Greater Glasgow and Clyde (NHSGGC) to promote safety and patient-centred care IV gentamicin is prescribed using specific prescribing, administering and monitoring (PAM) charts and restricted to 4 days treatment with provision of patient information leaflets (PILs).^2^ Medication incidents are common, consequently, IV gentamicin management is reviewed annually via point prevalence (PP) audits to support ongoing quality improvement (QI).^3^ In 2021, electronic prescribing (HEPMA) was introduced to NHSGGC, resulting in a change in the IV gentamicin prescribing process at ward level and prompting the need to assess the impact of this change.

**Objectives:**

To collect data to assess the impact of HEPMA introduction on the management of IV gentamicin at the Royal Alexandra Hospital (RAH) and identify target areas for improvement.

**Methods:**

An electronic data collection tool (Microsoft Forms) was developed, piloted, standardized and agreed. Daily HEPMA antimicrobial reports were used (Nov–Dec 2022) to identify RAH adult inpatients prescribed IV gentamicin. Prophylactic gentamicin prescriptions were excluded. HEPMA records and IV gentamicin PAM charts were reviewed, data collected in real time, collated and compared with pre-HEPMA data. Areas for improvement were identified and QI targets set.

**Results:**

In total 84 patients (55% male), mean age 68 years (range 20–94 years) were reviewed. The most common indications for IV gentamicin were intra-abdominal sepsis (31%) and urinary sepsis (24%). Reassuringly, IV gentamicin was appropriately prescribed on both the PAM chart and HEPMA in 96% of patients, there was a high standard of initial dose (96%) and frequency (100%) calculations and 96% of treatment durations were within the 4 day time window. A number of areas for targeted QI were identified. In total 21% of doses were delayed (>1 h from prescribed time) and only 32% of administered doses were documented on HEPMA. There was a reduction in initial gentamicin TDM samples taken at the correct time (63%) compared with pre-HEPMA median data (80%). Documentation of 48 h gentamicin on the PAM chart was correct in 82% of prescriptions. In 93% of PAM charts the PIL section was incomplete and in only 5% of patients was provision of a PIL and counselling documented. In total 59% of patients reviewed were considered suitable for IV gentamicin counselling with poor clinical condition and cognitive impairment the main barriers identified. Staff highlighted unclear responsibility and lack of time, resources, and training as barriers to providing IV gentamicin PILs and patient counselling.

**Conclusions:**

Following introduction of HEPMA IV gentamicin dose calculations and treatment duration reviews were of a high standard. A number of areas for targeted QI were identified including documentation of IV gentamicin dose administrations on HEPMA, dose delays, initial TDM sampling, documentation of 48 h prescribing and addressing the barriers identified to improve provision of IV gentamicin counselling and PILs in the acute hospital setting.
